# Late-Onset Bartter Syndrome Type II Due to a Novel Compound Heterozygous Mutation in *KCNJ1* Gene: A Case Report and Literature Review

**DOI:** 10.3389/fmed.2022.862514

**Published:** 2022-04-07

**Authors:** Mi Tian, Hui Peng, Xin Bi, Yan-Qiu Wang, Yong-Zhe Zhang, Yan Wu, Bei-Ru Zhang

**Affiliations:** ^1^Department of Nephrology, Shengjing Hospital of China Medical University, Shenyang, China; ^2^Guangzhou KingMed Center for Clinical Laboratory Co, Ltd., Guangzhou, China

**Keywords:** Bartter syndrome type II, *KCNJ1* gene mutation, nephrocalcinosis, hypokalemia, late onset

## Abstract

**Background:**

Bartter syndrome (BS) type II is a rare autosomal recessive renal tubular disorder caused by mutations in the *KCNJ1* gene, which encodes the apical renal outer medullary potassium (ROMK) channel in the thick ascending limb (TAL) of Henle’s loop. BS type II is typically considered as a disorder of infancy and seldom seen in adults.

**Case Presentation:**

A 34-year-old woman was admitted with generalized body numbness and hand convulsions, without growth retardation. Laboratory tests revealed hypokalemic metabolic alkalosis, hyperreninemic hyperaldosteronism, and nephrocalcinosis. She was misdiagnosed during the initial diagnosis process and was finally diagnosed with late-onset BS type II *via* genetic testing through next-generation sequencing combined with Sanger sequencing. A novel compound heterozygous p.Leu207Ile/p. Cys308Arg variant in exon 5 of the *KCNJ1* gene from her parents was identified and speculated to be a potential pathogenic gene variation.

**Conclusion:**

We report a case of late-onset BS type II with a novel compound heterozygous mutation in *KCNJ1*. Both variants are novel and have never been reported. Our report will have a significant impact on the diagnosis of BS in other patients without typical clinical presentations and emphasizes the importance of genetic investigation.

## Introduction

Bartter syndrome (BS) is a rare, autosomal recessive or dominant inheritance of salt-losing kidney disorder, with a prevalence of 1 in 1,000,000 (1). It is characterized by hypokalemia, metabolic alkalosis, hyperreninemia, hyperaldosteronism, hypercalciuria, polyuria, and polydipsia, accompanied by normal or low blood pressure (2). BS can be divided into several subtypes according to the mutations in different genes encoding the transporters involved in salt reabsorption in the thick ascending limb (TAL) of Henle’s loop (3). BS type I is caused by mutations in *SCL12A1*, which encodes the Na-K-2Cl cotransporter (NKCC2) in the apical membrane of epithelial cells in the TAL. BS type II is caused by mutations in *KCNJ1*, which encodes the renal outer medullary potassium (ROMK) channel. Potassium recycling through the ROMK channel to maintain the potassium concentrations in the lumina is crucial for proper NKCC2 function; thus, BS type I and type II have similar prenatal presentations (4). BS type III is associated with mutations in *CLCNKB*, which encodes the basolateral chloride channel CLC-Kb. BS type IV comprises two unique defects. BS type IVa is caused by mutations in the *BSND* gene encoding barttin, which is an essential subunit for CIC-Ka and CIC-Kb channels. However, BS type IVb is caused by digenic mutations in both *CLCNKB* and *CLCNKA*, which encode CLC-Kb and CLC-Ka channels, respectively (5). Two other subtypes of BS have been recently identified. (1) Transient neonatal BS is caused by mutations in melanoma-associated antigen D2 (*MAGED2*). In this type of BS, tubulopathy spontaneously improves within the first few months of life in survivors (6). (2) Autosomal dominant hypocalcemia is caused by gain-of-function mutations in the *CASR*, which encodes the calcium ion-sensing receptor (CaSR) in the basolateral cell membrane of the TAL.

Previously, another terminology was proposed to separate BS into “antenatal BS” (type I, II, and IV BS), associated with a more severe presentation, and “classic BS” (type III BS), associated with a later presentation in childhood. Type II BS is an antenatal/neonatal BS in which renal disorder begins in the utero, accounting for the polyhydramnios and premature delivery that is typical in affected infants. It can present life-threatening volume depletion due to severe renal salt wasting and characteristic BS clinical manifestations in the neonatal period, even resulting in stunted growth. This BS type likely represents a disorder in infancy but not in adulthood.

Here, we report an adult case with an unusually mild clinical presentation of BS known as late-onset BS type II, which contributes to a novel potential pathogenic compound heterozygous mutation in the *KCNJ1* gene in an Asian pedigree discovered through genetic testing.

## Case Presentation

A 34-year-old Chinese woman was admitted with the chief complaint of repeated generalized body numbness and hand convulsions experienced for the last 13 years, which have worsened in the 2 months. Thirteen years ago, she presented to the hospital with the same symptoms and had been told that she had hypokalemia and hypocalcemia, but she had not taken potassium chloride supplementation regularly and no follow-up after discharge. During these 13 years, she only took potassium chloride supplements when her symptoms worsened and stopped taking them as long as they were relieved. Occasional tests suggested that hypokalemia persisted. Her symptoms worsened 2 months before the latest hospitalization, and potassium chloride supplements did not significantly alleviate the symptoms. She presented to a local hospital and was informed that she had hypokalemia with a serum potassium level of 2.9 mmol/L, accompanied by hypomagnesemia and hypocalcemia. She was diagnosed with medullary sponge kidney (MSK) based on intravenous pyelography (IVP) examination (Supplementary Figure 1A) and renal tubular acidosis (RTA) type I by a local physician and was prescribed potassium citrate granules.

Although information on her antenatal course is not available, she was not a premature baby; she was delivered after a full-term pregnancy. There was no serious dehydration, electrolyte disorders or other symptoms that needed to be treated after birth. She developed normally, without any visible deformity, and had normal intellectual development. However, her parents revealed that she has experienced symptoms of thirst, polydipsia, and polyuria since childhood. Her medical history is unremarkable, and she has denied any medications, including diuretics and laxatives, alcohol, or other drugs. The parental family history is unremarkable, and she has no siblings. She is married and has one healthy child. No one else in the family experienced similar symptoms.

Her weight was 50 kg, height 162 cm, pulse 88/min, and blood pressure 130/80 mmHg. Physical examination results were normal.

Laboratory findings (Table 1) revealed hypokalemia with a serum potassium level of 3.43 mmol/L. In a 24-h urine collection, excretion of potassium was 88.36 mmol/d, suggesting renal potassium wasting as the source of hypokalemia. She had hypocalcemia with a calcium level of 2.03 mmol/L, but her 24-h urinary calcium excretion level was normal. She had hypomagnesemia with a serum magnesium concentration of 0.48 mmol/L. Arterial blood gas analysis showed a pH of 7.54, pCO_2_ of 31 mmHg, and HCO_3_ of 26.5 mmol/L, suggesting metabolic alkalosis. Urinalysis revealed a pH of 7 and specific gravity (SG) of 1.009. After 12 h of water deprivation, she had a reduced urine osmolality of 227 mOsm/kg at a plasma osmolality of 289 mOsm/kg, indicating impaired urine concentration function. There was proteinuria + - and no hematuria. However, her 24-h urinary protein quantification was 1.04 g/d. She also had hyperreninemia and hyperaldosteronism. The sodium and chloride levels were normal. Serum creatinine was 76.1 μmol/L [estimated glomerular filtration rate (eGFR) (CKD-EPI) 88.03 mL/min], indicating normal renal function. Her parathyroid hormone level was normal. Ultrasound examination showed medullary nephrocalcinosis or medullary sponge kidney in both kidneys (Supplementary Figure 1B).

**TABLE 1:** Laboratory investigations performed during the two hospital admissions.

Laboratory findings	First admission	Second admission	Normal values
Serum creatine, umol/L	62.4	76.1	59–104
Serum urea nitrogen, mmol/L	3.83	7.43	3–7.2
Plasma potassium, mmol/L	2.65	3.43	3.5–5.5
Plasma sodium, mmol/L	137.9	140	136–145
Plasma chlorine, mmol/L	94.8	105.5	96–108
Plasma calcium, mmol/L	1.97	2.03	2.1–2.55
Plasma phosphate, mmol/L	1.13	1.15	0.9–1.6
Plasma magnesium, mmol/L	0.59	0.48	0.67–1.15
Plasma parathyroid hormone, pg/mL	16.13	16.13	15–65
Urinary sodium, mmol/day	122	122	130–260
Urinary potassium, mmol/day	97.69	88.36	25–100
Urinary chlorine, mmol/day	301.3	148	170–250
Urinary calcium, mmol/day	10.95	6.84	2.5–7.5
Urinary phosphate, mmol/day	11.23	17.02	23–48
Serum renin, ng/mL	3.4	16.7	0.15–2.33
Serum Aldosterone, pg/mL	325	1038.7	30–160
Urinary pH	7.494	7	4.5–8.0
Urinary SG	1.009	1.009	1.003–1.030
Blood pH	7.494	7.54	7.35–7.45
Plasma bicarbonate, mmol/L	40.5	31	22–26

## Genetic Analysis

Clinical findings of recurrent hypokalemia with renal potassium wasting, metabolic alkalosis, hyperreninemia, and hyperaldosteronism raised the suspicion of BS or Gitleman syndrome (GS). Differential diagnosis depended on genetic testing.

Following informed consent, genomic deoxyribonucleic acid (DNA) of the patient was extracted from peripheral blood according to the manufacturer’s standard procedure using the QIAamp DNA Blood Midi Kit (Qiagen, Hilden, Germany). The extracted DNA was fragmented by DNase, purified using magnetic beads, amplified using polymerase chain reaction (PCR) and connected to the adapter sequence. The target area of the whole exome was captured and purified using an IDT XGen Exome Research Panel probe (IDT Corporation, United States). All the amplified libraries were subsequently sent to a high-throughput sequencing kit for sequencing on a NovaSeq 6000 sequencer (Illumina, San Diego, CA, United States). For the genetic analysis of *SLC12A1* (NKCC2), *KCNJ1* (ROMK), *CLCNKB* (ClC-Kb), *CLCNKA* (ClC-Ka), *BSND* (Barttin), *CASR* (CaSR), *MAGED2* (MAGE-D2), and *SLC12A3* (NCCT), which are responsible for BS or GS, gene exon coding regions and exon-intron junction regions were sequenced. Because MSK was also suspected in other hospitals, the reported potentially related genes according to the OMIM database, such as *MKS1, TMEM216, TMEM67, CEP290, RPGRIP1 L, CC2D2A, NPHP3, TCTN2, B9D1, B9D2, TMEM231, KIF14*, and *TMEM107*, were also analyzed.

We performed data analysis and bioinformatic processing to detect potential variants. We used the Burrows–Wheeler aligner (BWA) algorithm to compare all data with the reference sequence (UCSC hg19) (7) and the method in the reported literature to annotate the data (8). Data were screened by reference screening process (9). According to variant classification standards proposed by the American College of Medical Genetics and Genomics (ACMG) (10), the clinical significance of these variants was identified (9).

**FIGURE 1:**
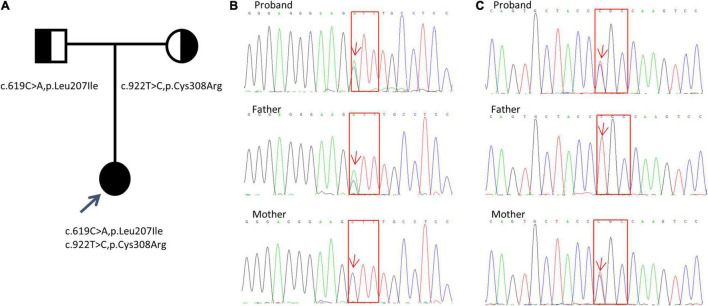
Detection of KCNJ1 variants in a Chinese family. Two novel heterozygous variants (c.619C > A, p.Leu207Ile and c.922T > C, p.Cys308Arg) were identified. **(A)** Pedigree of the family. **(B,C)** Sanger sequencing electropherograms (partial) of the patient and her parents are shown. The results of c.619C > A, p.Leu207Ile variant are shown in **(B)**, and those of the c.922T > C, p.Cys308Arg variant are shown in **(C)**. Red arrows indicate the positions of the variants. Red squares indicate the corresponding altered amino acids.

The analysis results showed a compound heterozygous missense mutation in exon 5 of the *KCNJ1* gene. The c.619C > A variant resulted in a change from a leucine codon to an isoleucine codon (p. Leu207Ile), and the c.922C > T variant resulted in a change from a cysteine codon to an arginine codon (p. Cys308Arg). To further prove the inheritance of the variation, we used the Sanger sequencing to examine the mutations in patient’s parents with consent, and discovered that c.619C > A was inherited from her father and c.922C > T was inherited from her mother (Figure 1). Based on the clinical and genetic findings, the patient was diagnosed with late-onset BS type II.

## Discussion

Historically, BS has been categorized by phenotypic characteristics, such as antenatal BS and classic BS, based on age and severity of presentation. However, emerging data show a wide spectrum of severity in all forms of BS; some patients with type I, II, or IV BS present with late-onset form (11), whereas some patients with type III BS may appear with a severe antenatal presentation (12), although relatively rare. Type II BS is classified as antenatal BS. Most patients are born prematurely and present life-threatening volume depletion and electrolyte disorders. If they survive in the neonatal period, they will show growth retardation or even abnormal development. However, a few patients with type II BS can spontaneously manifest transient hyponatremia of prematurity with complete resolution and only demonstrate persistent polyuric and hypercalciuria without the development of nephrocalcinosis (13). To date, six cases of late-onset BS type II have been reported (14–19). All these cases developed normally and were finally diagnosed in adulthood because of various mild clinical symptoms as reported in Table 2. These cases strongly suggest that BS is a differential disease that should be considered in adults with similar manifestations, and it requires timely genetic testing. However, it has been reported that a genetic diagnosis cannot be established in approximately two-thirds of patients with adult-onset BS (4).

**TABLE 2:** Summary of clinical information and gene mutations of six reported cases of late-onset BS type II and our case.

No	1	2	3	4	5	6	7
Author	Sharma (14)	Huang (15)	Gollasch (16)	Li (17)	Yaqub (18)	Elferl (19)	Present case
Sex	Female	Male	Female	Female	Male	Male	Female
Age of presentation	8.5	35	43	34	38	26	34
Clinical presentation of admission	Polyuria, polydipsia	Lower back pain attribute to nephrocalcinosis	Nephrocalcinosis	Weakness	Fatigue, lethargy, lower limb weakness	Generalized weakness	Generalized body numbness, hands convulsion
Previous clinical manifestations	Polyuria, polydipsia	No	Thirst, polyuria	Polyuria, polydipsia	Bilateral flank pain, polyuria	Thirst, polydipsia, polyuria	Thirst, polydipsia, polyuria
Perinatal period abnormalities	No	Unknown	No	Polyhydramnios	Unknown	Unknown	No
Family history	No	No	No	No	Unknown	No	No
Nephrocalcinosis	(+)	(+)	(+)	(+)	(+)	(+)	(+)
Serum potassium level (mmol/L)	2.5	2.8	3	2.4	1.5	1.7	3.43
Hyperreninemia/ hyperaldosteronemia	(+)	(+)	(+)	(+)	(+)	(+)	(+)
Urine potassium	Increase	Normal	ND	Increase	Increase	Increase	Increase
Serum calcium	Normal	Decrease	Normal	Normal	Decrease	Normal	Decrease
24 h urine calcium	Increase	Increase	Increase	Increase	Increase	Increase	Normal
Serum creatinine (μ mol/L)	44	122	97	105	203	96	76.1
Serum magnesium	Normal	ND	Normal	Normal	Normal	Normal	ND
Mutation	Heterozygous	Homozygous	Heterozygous	Heterozygous	ND	Homozygous	Heterozygous
DNA sequence change	c.268G > T c.632T > G	c.658C > T	c.197T > A c.875G > A	c.701C > T c.212C > T	ND	c.658C > T	c.619C > A c.922C > T
Amino acid change (parent)	p.Gly90Trp(M) p.Ile211Ser(F)	p.Leu220Phe(ND)	p.Ile66Asn(ND) p.Arg292Gln(ND)	p.The234Ile(M) p.Thr71Me(F)	ND	p.Leu220Phe(ND)	p. Leu207Ile(F) p.Cys308Arg(M)

*ND, not determined; M, mother; F, father.*

**FIGURE 2:**
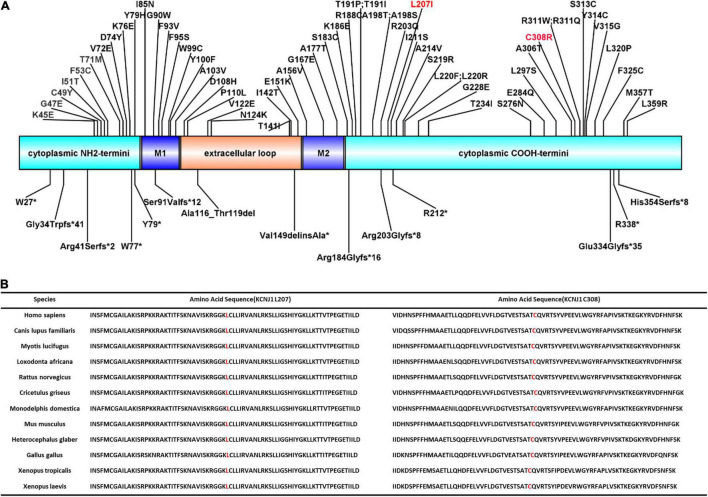
ROMK protein structure and conservation of L207 and C308 among homologs. **(A)** Domains of the protein with reported mutations associated with type II Bartter syndrome. M1 and M2 represent two transmembrane domains. Red font indicates the mutations reported in this study. **(B)** Complete preservation of both the leucine amino acid (L207) and cysteine amino acid (C308) among homologs through Xenopus laevis.

*KCNJ1* is located on chromosome 11q24 and consists of five exons, with exon 5 encoding most of the sequences of the protein channel and being the most influential putative functional domain of *KCNJ1*. More than 70 *KCNJ1* mutations have been described to date (Figure 2A), most of which are missense or non-sense mutations substituting conserved amino acid residues, predominantly within coding exon 5 (20). The five reported late-onset BS type II cases discovered through genetic tests had different mutations in *KCNJ1*, but all were in exon 5. Among these, only two cases presented with a compound heterozygous mutation. In this study, we identified a novel compound heterozygous mutation (p.Leu207Ile/p. Cys308Arg), which has not been reported previously. Next generation sequencing-based mutation screening combined with Sanger sequencing has been proven to be a reliable method for molecular diagnosis. These variants were not included in the large public population database gnomAD^1^, indicating that they are extremely rare. Although we did not directly verify the effect of heterozygous mutations on protein function, we hypothesized that they are likely to have a significant impact. PolyPhen-2 and Scale Invariant Feature Transform (SIFT) software predicted that both mutations were deleterious. These positions are entirely conserved among species (Figure 2B), indicating that the mutations severely impair the function of the protein. Furthermore, the combination of mutations in a compound heterozygous state may not be arbitrary but may influence the tertiary or quaternary structure of proteins and further determine the severity of the phenotype (21). Thus, the compound heterozygous state of Leu207Ile and Cys308Arg may have partial intrinsic transport, accounting for the late onset.

Gitleman syndrome is also a rare recessive salt-losing tubulopathy that results in impaired salt reabsorption, caused by mutations in the *SLC12A3* gene encoding NaCl cotransporter in distal convoluted tubules (DCT). Although variants of BS and GS are genotypically distinct, there is a considerable overlap in their clinical presentation, such as hypokalemia due to renal potassium loss, normal blood pressure, and metabolic alkalosis, as observed in this case. BS shows an earlier age of onset and heavier clinical symptoms than GS, and patients with BS often have hypercalciuria, whereas GS is often accompanied by hypomagnesemia, low urinary calcium excretion and normal urine volume. In our patient, the late onset, no growth retardation, no hypercalciuria, and hypomagnesemia indicated a diagnosis of GS, but polyuria and impaired urinary concentration function could not be explained by GS. Of course, genetic investigation is fundamental for differential diagnosis between BS and GS. The patient was finally diagnosed with BS, but no GS. In fact, hypomagnesemia is more common in GS and one of important differences between GS and BS. The mechanism of hypomagnesemia is through downregulation of apically located transient receptor potential channel subfamily M, member 6 (TRPM6), a magnesium-permeable channel predominantly expressed in the DCT (22). However, it has been reported that 20-30% of patients with BS also have hypomagnesemia, and the mechanism is unclear. The urinary calcium level is high in BS but low in GS, however, the patient has normal urinary calcium excretion in this administration, which is inconsistent with BS and cannot explain her nephrocalcinosis. However, hypercalciuria in the patient was identified by the test 13 years ago. Nephrocalcinosis is common in patients with BS, which is presumed to be due to their hypercalciuria and can be observed after some weeks of severe hypercalciuria (23). As in a previous reported case of BS, renal ultrasound on day 5 was normal, but on day 27, it showed a thin hyperechoic rim in the pyramids suggestive of nephrocalcinosis (24). The reason for the change in the urinary calcium level in this patient from high to normal is unclear. The fact that nephrocalcinosis is found in all patients with late-onset BS suggests that hypercalciuria will exist in all cases of BS type II in the early stage, although different levels of urinary calcium excretion will occur at the time of diagnosis.

Although the characteristic hypokalemic metabolic alkalosis was not found in MSK, the changes in renal imaging and common clinical manifestations such as urinary concentration dysfunction, thirst, polydipsia, and polyuria led to diagnosis of MSK, and further hypokalemia was attributed to type I renal tubular acidosis, a common complication of MSK. MSK is a rare congenital malformation defined as the dilatation of the medullary and papillary portions of the collecting ducts due to cystic damage to the distal nephron. The disease is often sporadic and rarely presents with familial inheritance in an autosomal dominant manner (25). The diagnosis of MSK mainly depends on imaging examinations. Currently, IVP is still regarded as the gold standard for diagnosis. Metabolic alkalosis and the clinical finding of hyperreninemic hyperaldosteronism, combined with genetic analysis, ruled out MSK. However, as one of several common causes of medullary nephrocalcinosis, MSK should be a differential diagnosis of BS.

The patient had increased urinary protein levels, with the 24-h urinary protein level of 1.04 g/d. Albumin and immunoglobulin were mainly increased, and urinary a1 microglobulin was normal, suggesting glomerular proteinuria, which could not be explained by tubulopathy. A renal biopsy could not be performed because the patient had nephrocalcinosis. However, there have been reports of patients who develop focal segmental glomerulosclerosis during the course of BS due to chronic stimulation of the renin-angiotensin system with secondary chronic glomerular hyperfiltration (26). At present, prognosis in many cases of BS is good, and the kidney rarely enters the dialysis stage; however, there are few long-term follow-up cases. The interval between the two hospitalizations of this patient was 13 years, and the renal function remained stable, which also suggests that the renal prognosis was good in late-onset BS. However, it is not clear whether an increase in urinary protein levels affects the progression of renal disease.

## Conclusion

We report a special late-onset BS type II case with a novel compound heterozygous mutation in the *KCNJ1* gene. Our study suggests a high degree of variability in aBS II regarding disease severity and emphasizes the importance of timely genetic testing for individuals with suspected diseases. Although gene monitoring has enhanced the convenience and accuracy of BS diagnosis, the disease remains easily missed or misdiagnosed. Case reports such as this study are expected to improve our understanding and diagnosis of BS. Further studies are required to verify the effects of this novel gene mutation.

## Ethics Statement

The studies involving human participants were reviewed and approved by the China Medical University. The patients/participants provided their written informed consent to participate in this study. Written informed consent was obtained from the individual(s) for the publication of any potentially identifiable images or data included in this article.

## Author Contributions

MT contributed to data collection and manuscript writing. B-RZ supervised the data collection and finalized the manuscript. HP, Y-QW, Y-ZZ, and YW contributed to data collection. XB participated in the data collection and analysis. All authors contributed to the article and approved the submitted version.

## Conflict of Interest

XB was employed by Guangzhou KingMed Center for Clinical Laboratory Co, Ltd. The remaining authors declare that the research was conducted in the absence of any commercial or financial relationships that could be construed as a potential conflict of interest.

## Publisher’s Note

All claims expressed in this article are solely those of the authors and do not necessarily represent those of their affiliated organizations, or those of the publisher, the editors and the reviewers. Any product that may be evaluated in this article, or claim that may be made by its manufacturer, is not guaranteed or endorsed by the publisher.

## Footnotes

1
https://gnomad.broadinstitute.org/

## Supplementary Material

Supplementary Figure 1 Kidney imaging findings of intravenous pyelography and ultrasound. **(A)** Intravenous pyelography shows “bouquet of flowers” appearance of the dilated tubules within the renal medulla of both kidneys. **(B)** Renal ultrasonography shows multiple irregular hyperechoic stones in the pyramids of both kidneys.

## References

[1] Ji W Foo JN O’Roak BJ Zhao H Larson MG Simon DB Rare independent mutations in renal salt handling genes contribute to blood pressure variation. *Nat Genet.* (2008) 40:592–9. 10.1038/ng.118 18391953 PMC3766631

[2] Seyberth HW Weber S Komhoff M. Bartter’s and Gitelman’s syndrome. *Curr Opin Pediatr.* (2017) 29:179–86. 10.1097/MOP.0000000000000447 27906863

[3] Cunha TDS Heilberg IP. Bartter syndrome: causes, diagnosis, and treatment. *Int J Nephrol Renovasc Dis.* (2018) 11:291–301. 10.2147/IJNRD.S155397 30519073 PMC6233707

[4] Mrad FCC Soares SBM de Menezes Silva LAW Dos Anjos Menezes PV Simoes ESAC. Bartter’s syndrome: clinical findings, genetic causes and therapeutic approach. *World J Pediatr.* (2021) 17:31–9. 10.1007/s12519-020-00370-4 32488762

[5] Komhoff M Laghmani K. Pathophysiology of antenatal Bartter’s syndrome. *Curr Opin Nephrol Hypertens.* (2017) 26:419–25. 10.1097/MNH.0000000000000346 28598867

[6] Laghmani K Beck BB Yang SS Seaayfan E Wenzel A Reusch B Polyhydramnios, transient antenatal Bartter’s syndrome, and MAGED2 mutations. *N Engl J Med.* (2016) 374:1853–63. 10.1056/NEJMoa1507629 27120771

[7] Li H Durbin R. Fast and accurate long-read alignment with burrows-wheeler transform. *Bioinformatics.* (2010) 26:589–95. 10.1093/bioinformatics/btp698 20080505 PMC2828108

[8] Zhang L Zhang J Yang J Ying D Lau YL Yang W. PriVar: a toolkit for prioritizing SNVs and indels from next-generation sequencing data. *Bioinformatics.* (2013) 29:124–5. 10.1093/bioinformatics/bts627 23104884

[9] Yang Y Muzny DM Reid JG Bainbridge MN Willis A Ward PA Clinical whole-exome sequencing for the diagnosis of mendelian disorders. *N Engl J Med.* (2013) 369:1502–11. 10.1056/NEJMoa1306555 24088041 PMC4211433

[10] Bahcall OG. Genetic testing. ACMG guides on the interpretation of sequence variants. *Nat Rev Genet.* (2015) 16:256–7. 10.1038/nrg3940 25854183

[11] Pressler CA Heinzinger J Jeck N Waldegger P Pechmann U Reinalter S Late-onset manifestation of antenatal Bartter syndrome as a result of residual function of the mutated renal Na+-K+-2Cl- co-transporter. *J Am Soc Nephrol.* (2006) 17:2136–42. 10.1681/ASN.2005101071 16807401

[12] Seys E Andrini O Keck M Mansour-Hendili L Courand PY Simian C Clinical and genetic spectrum of bartter syndrome type 3. *J Am Soc Nephrol.* (2017) 28:2540–52. 10.1681/ASN.2016101057 28381550 PMC5533235

[13] Verma S Chanchlani R Siu VM Filler G. Transient hyponatremia of prematurity caused by mild Bartter syndrome type II: a case report. *BMC Pediatr.* (2020) 20:311. 10.1186/s12887-020-02214-6 32590952 PMC7318402

[14] Sharma A Linshaw MA. A novel compound heterozygous ROMK mutation presenting as late onset Bartter syndrome associated with nephrocalcinosis and elevated 1,25(OH)(2) vitamin D levels. *Clin Exp Nephrol.* (2011) 15:572–6. 10.1007/s10157-011-0431-3 21431899

[15] Huang L Luiken GP van Riemsdijk IC Petrij F Zandbergen AA Dees A. Nephrocalcinosis as adult presentation of Bartter syndrome type II. *Neth J Med.* (2014) 72:91–3. 24659592

[16] Gollasch B Anistan YM Canaan-Kuhl S Gollasch M. Late-onset Bartter syndrome type II. *Clin Kidney J.* (2017) 10:594–9. 10.1093/ckj/sfx033 28979772 PMC5622898

[17] Li J Hu S Nie Y Wang R Tan M Li H A novel compound heterozygous KCNJ1 gene mutation presenting as late-onset Bartter syndrome: case report. *Medicine (Baltimore).* (2019) 98:e16738. 10.1097/MD.0000000000016738 31441846 PMC6716717

[18] Yaqub S Arif MS. A case of Bartter’s syndrome presenting in adulthood. *Iran J Kidney Dis.* (2020) 14:65–7. 32156844

[19] Elfert KA Geller DS Nelson-Williams C Lifton RP Al-Malki H Nauman A. Late-onset Bartter syndrome type II due to a homozygous mutation in KCNJ1 gene: a case report and literature review. *Am J Case Rep.* (2020) 21:e924527. 10.12659/AJCR.924527 32997650 PMC7534490

[20] Stenson PD Mort M Ball EV Chapman M Evans K Azevedo L The human gene mutation database (HGMD((R))): optimizing its use in a clinical diagnostic or research setting. *Hum Genet.* (2020) 139:1197–207. 10.1007/s00439-020-02199-3 32596782 PMC7497289

[21] Welling PA Ho K. A comprehensive guide to the ROMK potassium channel: form and function in health and disease. *Am J Physiol Renal Physiol.* (2009) 297:F849–63. 10.1152/ajprenal.00181.2009 19458126 PMC2775575

[22] Nijenhuis T Vallon V van der Kemp AW Loffing J Hoenderop JG Bindels RJ. Enhanced passive Ca2+ reabsorption and reduced Mg2+ channel abundance explains thiazide-induced hypocalciuria and hypomagnesemia. *J Clin Invest.* (2005) 115:1651–8. 10.1172/JCI24134 15902302 PMC1090474

[23] Garnier A Dreux S Vargas-Poussou R Oury JF Benachi A Deschenes G Bartter syndrome prenatal diagnosis based on amniotic fluid biochemical analysis. *Pediatr Res.* (2010) 67:300–3. 10.1203/PDR.0b013e3181ca038d 19915517

[24] Gomez de la FC Novoa PJ Caviedes RN. Bartter syndrome: an infrequent tubulopathy of prenatal onset. *Rev Chil Pediatr.* (2019) 90:437–42. 10.32641/rchped.v90i4.932 31859717

[25] Fabris A Anglani F Lupo A Gambaro G. Medullary sponge kidney: State of the art. *Nephrol Dial Transplant.* (2013) 28:1111–9. 10.1093/ndt/gfs505 23229933

[26] Su IH Frank R Gauthier BG Valderrama E Simon DB Lifton RP Bartter syndrome and focal segmental glomerulosclerosis: a possible link between two diseases. *Pediatr Nephrol.* (2000) 14:970–2. 10.1007/s004670050054 10975308

