# Supplementary figures and images for: Late-Onset Bartter Syndrome Type II Due to a Novel Compound Heterozygous Mutation in KCNJ1 Gene: A Case Report and Literature Review
Source: Front Med (Lausanne). 2022 Apr 7;9:862514. doi: 10.3389/fmed.2022.862514 (PMC9021870; doi:10.3389/fmed.2022.862514)

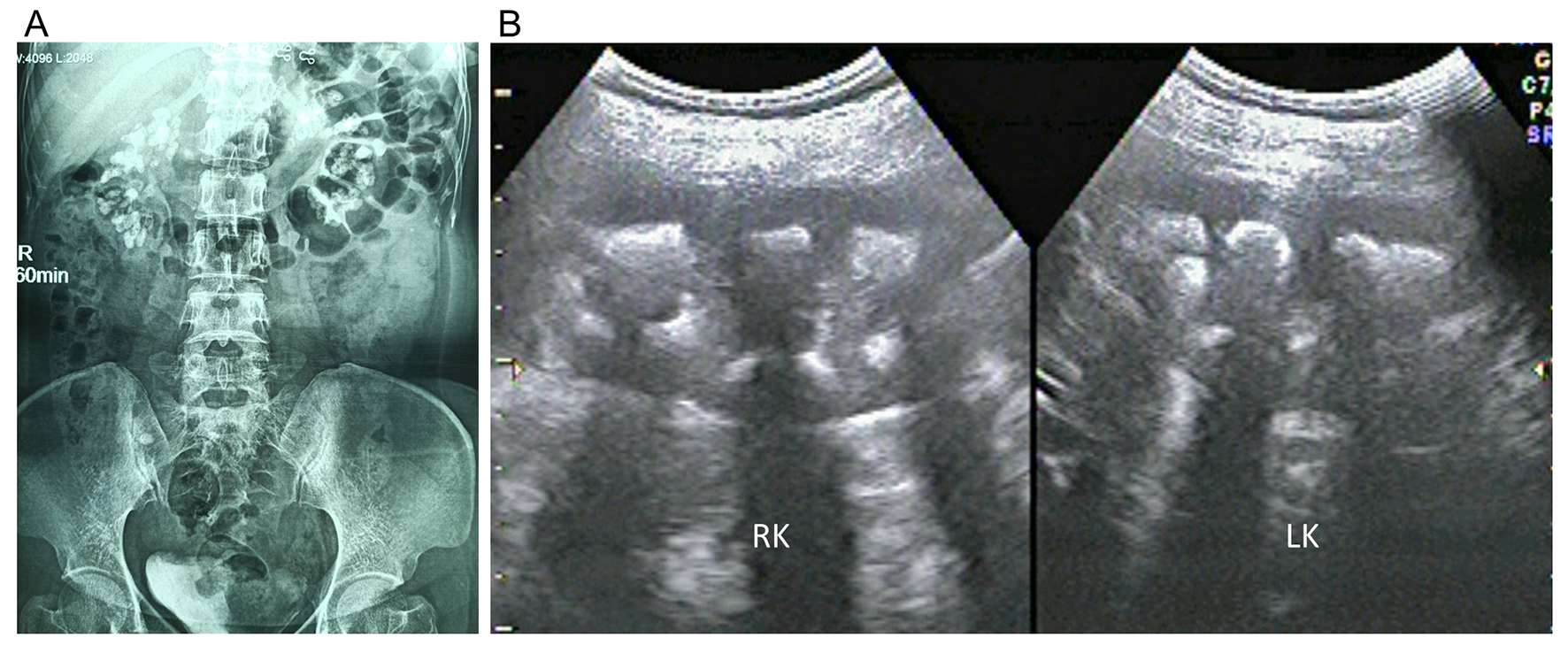

Supplement: Supplementary Figure 1 — Kidney imaging findings of intravenous pyelography and ultrasound. (A) Intravenous pyelography shows “bouquet of flowers” appearance of the dilated tubules within the renal medulla of both kidneys. (B) Renal ultrasonography shows multiple irregular hyperechoic stones in the pyramids of both kidneys. [file Image_1.tif]
